# Genetic Manipulation as a Tool to Unravel *Candida parapsilosis* Species Complex Virulence and Drug Resistance: State of the Art

**DOI:** 10.3390/jof7060459

**Published:** 2021-06-07

**Authors:** Marina Zoppo, Noemi Poma, Mariagrazia Di Luca, Daria Bottai, Arianna Tavanti

**Affiliations:** 1 Department of Environmental Toxicology, Eawag, Swiss Federal Institute of Aquatic Science and Technology, 8600 Dübendorf, Switzerland; marina.zoppo@eawag.ch; 2 Department of Chemistry and Industrial Chemistry, University of Pisa, 56127 Pisa, Italy; n.pomasajama@studenti.unipi.it; 3 Department of Biology, University of Pisa, 56127 Pisa, Italy; mariagrazia.diluca@unipi.it (M.D.L.); daria.bottai@unipi.it (D.B.)

**Keywords:** *Candida parapsilosis* species complex, genetic manipulation, SAT1-flipper cassette, CRISPR/Cas9 system, virulence, drug resistance

## Abstract

An increase in the rate of isolation of *Candida parapsilosis* in the past decade, as well as increased identification of azole-resistant strains are concerning, and require better understanding of virulence-like factors and drug-resistant traits of these species. In this regard, the present review “draws a line” on the information acquired, thus far, on virulence determinants and molecular mechanisms of antifungal resistance in these opportunistic pathogens, mainly derived from genetic manipulation studies. This will provide better focus on where we stand in our understanding of the *C. parapsilosis* species complex–host interaction, and how far we are from defining potential novel targets or therapeutic strategies—key factors to pave the way for a more tailored management of fungal infections caused by these fungal pathogens.

## 1. Introduction

In recent years, we witnessed an increase in the frequency of invasive fungal infections worldwide and a significant change in the epidemiological scenario: if *Candida albicans* dominated the clinical setting over the last century, now non-*albicans Candida* (NAC) species are on the rise [1,2].

Among these, the *Candida* species, belonging to the *C. parapsilosis* complex, namely *Candida parapsilosis*, *C. orthopsilosis* and *C. metapsilosis* [3], are isolating in increasing rates, with *C. parapsilosis* currently representing the second most commonly isolated *Candida* species from blood cultures worldwide [4,5,6]. *C. parapsilosis* is considered a commensal yeast commonly found on human skin, but it is also known for nosocomial spread by hand carriage and because of its propensity to form biofilms on catheters and other medical or prosthetic devices. These aspects make *C. parapsilosis* of special concern in hospitalized patients and in immunocompromised individuals. In addition, *C. parapsilosis* is recognized as affecting newborns in neonatal intensive care units, accounting for 33% of all invasive *Candida* diseases, and for approximately 80% of NAC-associated infections in premature newborns [7,8,9]. Despite *Candida orthopsilosis* being less frequently isolated, evidence derived from in vitro and in vivo studies reveal that this species shares similar virulence traits and the ability to sustain serious infection to that of the most successful species of the complex [10]. *C. metapsilosis* is the least pathogenic species of the three, although it circulates in the nosocomial environment with various frequencies, according to different geographical areas [11].

Further complication in the management of invasive infections, due to the *C. parapsilosis* species complex, involves reduced susceptibility to echinocandins [12,13], considered as a first choice in the treatment of disseminated candidiasis, and the increasing isolation frequency of azole-resistant strains [14,15,16,17,18,19].

Comparative genomic analyses in these species have identified the presence of virulence-related gene families, such as aspartyl proteinases, lipases, or agglutinin-like sequence genes, whose role in virulence and pathogenicity is only starting to be elucidated [2,20,21,22,23]. The need to shed light on the molecular aspects underlying drug resistance phenotypes is derived from the clinical setting, where azole-resistant *C. parapsilosis* or *C. orthopsilosis* isolates are now being collected with increasing frequencies [19,24,25,26,27,28].

Given the increasing clinical relevance of the *C. parapsilosis* species complex, the substantial gap existing in our knowledge on their virulence properties or drug resistance determinants is concerning. To address this, the present review draws a line on the information acquired, thus far, on virulence and drug resistance in these opportunistic pathogens, mainly derived from gene disruption or gene editing studies, and to pave the way for better understanding and more tailored management of fungal infections caused by the *C. parapsilosis* species complex.

## 2. History of Genetic Manipulation in *C. parapsilosis*

**Figure 1:**
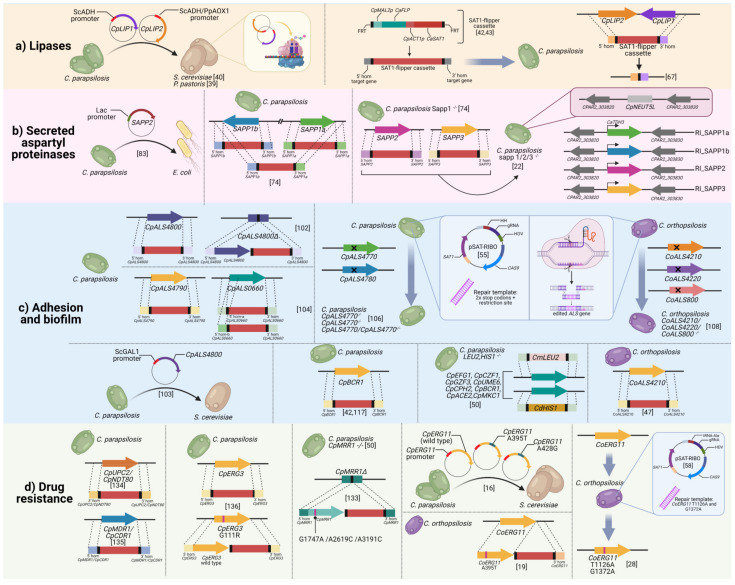
Overview of the genetic manipulation techniques employed to investigate the role of virulence and drug resistance genes in the *C. parapsilosis* species complex. The figure is divided into four panels describing the gene manipulation experiments performed to dissect the role of (**a**) lipases; (**b**) secreted aspartyl proteinases; (**c**) adhesion and biofilm; and (**d**) drug resistance genes, respectively. Each strategy illustrated in each color-coded panel is linked to its respective reference. A detailed description of each study is provided in the text of the review. Color-coding was applied to distinguish *C. parapsilosis* (green), *C. orthopsilosis* (violet), and *S. cerevisiae*/*P. pastoris* (brown). *ScADH*: *S. cerevisiae* alcohol dehydrogenase promoter; *PpAOX1 P. pastoris* methanol inducible promoter of the alcohol oxidase 1 gene; ScGAL1: galactose-inducible promoter; *CmLEU2*: *C. maltosa LEU2*; *CdHIS1*: *C. dubliniensis HIS1*. Figure created with BioRender (https://biorender.com/ (accessed on 7 June 2021)).

Before the development of gene manipulation platforms, genetic analysis of *C. parapsilosis* mainly relied on the use of parasexual genetic techniques [29], consisting in the generation of auxotrophic strains following ultraviolet (UV) irradiation, or on DNA fingerprinting approaches, such as pulsed-field gel electrophoresis (PFGE) [30], randomly amplified polymorphic DNA (RAPD) methods [31,32], DNA sequencing of internally transcribed spacer (ITS) regions [31], and restriction fragment length polymorphisms (RFLPs) analysis [33,34]. Genetic studies in *C. parapsilosis* have been hampered due to the absence of suitable genetic manipulation tools coupled with the lack of a sexual cycle and the diploid nature of its genome. Further steps towards a deeper understanding of *C. parapsilosis* biology and pathogenesis were achieved with the development of a genetic manipulation system based on the jointly usage of an autonomous replicating plasmid carrying the *CpGAL1* nutritional marker and a galactose auxotrophic strain of *C. parapsilosis* [35]. As reported by the same authors, introduction of the cloning vector inside *C. parapsilosis* was achieved through the establishment of a lithium acetate electroporation protocol, which was further optimized in the following years by other research groups to enable increasing transformation efficiencies [36,37]. Three years later, a dominant transformation system based on the *C. albicans IMH3* (*CaIMH3*) drug-resistant marker, conferring resistance to mycophenolic acid (MPA), was optimized by Gacser and colleagues, making gene manipulation of clinical isolates of *C. parapsilosis* finally possible [36]. Amplification of the MPA resistance gene was detected in all the *C. parapsilosis* transformants, while southern blot analysis indicated single and multiple integrations of the drug resistance gene either randomly or in a hot spot of the *C. parapsilosis* genome [36]. A further set of vectors based on auxotrophic (*CpGAL1*, *CpURA3*, *CpMET2*, *CpLYS4*) and dominant (*CaIMH3*) selection was developed in 2007 for genetic manipulation of *C. parapsilosis* [38]. Heterologous expression of *Candida* proteins in model yeasts such as *Saccharomyces cerevisiae* or *Pichia pastoris* has represented a powerful tool for protein function studies, aimed, for example, at investigating the catalytic activity associated with *C. parapsilosis* lipases [39,40]. The usage of yeast models as a surrogate host to express *Candida* proteins, however, has its own limitations, first and foremost, the mistranslation of the CUG codon into leucine instead of serine that occur when the recombinant proteins of the *C. parapsilosis* species complex are expressed in a model organisms outside the CTG clade [41]. A turning point for targeted gene disruption studies is represented by the work of Ding and Butler, with the adaptation for *C. parapsilosis* of the SAT1-flipper cassette system, a gene disruption cassette originally developed for *C. albicans* by Morschhäuser and colleagues [42,43] (Figure 1). In the original form, the SAT1-flipper cassette relies on the use of a dominant selection marker (*CaSAT1*), conferring resistance to the antimicrobic nourseothricin, under the control of the constitutive *C. albicans ACT1* promoter. The cassette contains a *C. albicans*-adapted flippase gene (*FLP*), which allows the recycle of the cassette and the inactivation of the remaining wild type allele through the recognition of two short flippase recognition target (*FRT*) sites located at both sides of the SAT-flipper cassette. The presence of the *C. albicans MAL2* promoter placed in front of the *FLP* gene ensures temporal control of gene expression and, therefore, the excision of the disruption cassette under inducible conditions. At both sites of the disruption cassette, two homology regions are cloned in order to allow the cassette integration in the locus of interest. Adaptation of the SAT1-flipper cassette for *C. parapsilosis* gene disruption involved the replacement of the *CaACT1* and *CaMAL2* promoter regions with the equivalent species-specific sequences. As a proof of principle, the Cp*URA3* gene, encoding for an enzyme taking part to the synthesis of pyrimidine ribonucleotides, and Cp*BRC1* gene, encoding for a transcription factor involved in biofilm formation, were knocked-out [42]. Since then, the SAT1-flipper cassette has been intensively used to interrogate *C. parapsilosis* gene function through the creation of null mutant and complemented strains, respectively through the sequential inactivation of both alleles of the target gene and the reintegration of a wild type copy in the resulting knockout strain. Restoration of the gene of interest can be performed through its integration in the original genomic location or in a neutral locus, such as the *CpNEUT5L* of *C. parapsilosis* [44], the ortholog of a large intergenic region of *C. albicans* (*NEUT5L*) commonly used for knock-in experiments [45,46]. Gene disruption using the SAT1-flipper cassette was also employed for the efficient generation of mutant strains in the closely related species, *C. orthopsilosis* [47]. Despite its widespread use, the SAT1-flipper also suffers from several drawbacks, which mainly depend on the laborious work for the cloning of the targeted gene homology regions and on the time-consuming process required for the generation of mutants due to the consecutive targeting of two alleles and the consequent recycle of the cassette. These impediments become insurmountable when interested in studying a large set of genes or when dealing with gene families. A compromise for such necessity was met with the development of gene disruption cassettes generated by fusion PCR. This technique, firstly developed in *S. cerevisiae* [48] and later adapted by Noble and colleagues in *C. albicans* [49], makes use of auxotrophic strains and disruption cassettes, bearing nutritional markers, rapidly assembled by fusion PCR. Generation of null mutants is achieved by auxotrophic complementation obtained via the sequential targeting of each allele with a different nutritional marker. This approach was also used in *C. parapsilosis* for the construction of a large-scale gene deletion collection carrying mutations in 100 genes encoding for transcription factors, protein kinases, and species-specific proteins [50]. Despite the high-throughput gene deletion analysis that this system offers, gene manipulation is still hampered by several hurdles. First, as for the SAT1-flipper cassette, PCR-synthesis of marker cassettes relies on two transformation steps for the inactivation of both alleles. Second, auxotrophic parental strains need to be used for the construction of null mutants, thus preventing gene function studies in clinical isolates. To conclude, generation of multiple knockouts in the same strain is unfeasible as the nutritional markers are not recycled. The development of the Clustered Regularly Interspaced Short Palindromic Repeats (CRISPR)/CRISPR-associated protein 9 (Cas9) technology as gene editing tool marked a revolutionary breakthrough in genetic manipulation, making the generation of mutants no longer an experimental bottleneck [51]. In the type II CRISPR/Cas9 system, 20 nucleotides at the 5′ end of the guide RNA (gRNA) directs an RNA-dependent endonuclease, called Cas9, to the genomic region of interest, using DNA-RNA complementary rules. The perfect complementarity and the presence of a protospacer adjacent motif (PAM) (5′-NGG) immediately after the target sequence, triggers Cas9 to operate a double strand break (DSB). The break is repaired either by the non-homologous end-joining (NHEJ), or homology-directed repair (HDR), resulting in the insertion/deletions (indels) of random DNA bases or in the precise insertion of specific sequences, respectively [52]. The simplicity and versatility of the CRISPR/Cas9 technology has drawn considerable attention over the years, resulting in the publication of countless works on gene editing platforms. The first application of CRISPR/Cas9 gene editing in *C. albicans* was reported by Vyas and colleagues in 2015 through the establishment of an integrative strategy where both a CTG codon-optimized version of Cas9 and a gRNA were integrated in the *C. albicans* genome, allowing the generation of single and multiple knockouts in a single transformation experiment [53]. Following this first report, the CRISPR/Cas9 method was further improved and adapted in other non-*albicans Candida* species (reviewed in [54]). For example, in 2017, the CRISPR/Cas9 system was adapted and further implemented for the first time in *C. parapsilosis* by Lombardi and co-workers through the establishment of a plasmid-based gene editing approach [55]. In this system, both Cas9 and the gRNA are maintained on a episomal vector, pRIBO, encompassing the dominant selection marker *SAT1* and an autonomously replicating sequence from *C*. *parapsilosis* (*ARS7,* [35]), which eliminates the need for genome integration of the CRISPR/Cas9 elements. Higher expression levels of the gRNA are ensured by the usage of an RNA polymerase II promoter together with the hammerhead (HH) and hepatitis delta virus (HDV) ribozymes [56,57] respectively cloned upstream and downstream the gRNA sequence. The co-transformation of pRIBO plasmid, along with a repair template flanked by 40-bp homology regions, allowed to efficiently generate gene deletions or incorporate unique tags in both reference and clinical isolates [55]. Moreover, in the absence of selection, the CRISPR vector is easily lost, allowing to sequentially target more genes in the same strain of interest. The episomal CRISPR/Cas9 strategy also proved to work for the generation of mutant strains in *C. orthopsilosis* [47]. A further improvement of the episomal CRISPR/Cas9 strategy described for *C. parapsilosis* was published by the same authors in 2019 with the replacement of the HH ribozyme with the *C. parapsilosis* tRNA^Ala^ sequence. This substitution allowed not only to accelerate the cloning procedure and speed up the generation of mutant strains [58], but also to achieve a more efficient gRNA maturation thanks to the endogenous ribonuclease Z-mediated cleavage of the tRNA [57,58]. In the same study, *C. parapsilosis* heterozygous strains were obtained either by varying the distance between the Cas9 DSB site and the inserted DNA or via transformation with two different repair templates. Both the pRIBO plasmid approach and the tRNA strategy, were used for the efficient generation of mutant strains in the closely related species, *C. orthopsilosis* and *C. metapsilosis* [28,58] demonstrating, once again, the versatile applicability of genetic manipulation systems among members of the same complex.

## 3. Characterization of *C. parapsilosis* Species Complex Virulence Factors

The shift from non-pathogenic commensal microorganism to pathogen is the result of a delicate misbalance in the interplay between the yeast and the host, which is facilitated by the expression of a variety of virulence factors such as hydrolytic enzymes, adhesins, and biofilm formation, contributing to the pathogenesis of the *Candida parapsilosis* species complex. The availability of sequenced genomes [21,59,60,61], together with the development of diverse and more optimized genetic manipulation techniques, has allowed the publication of several studies aimed at dissecting the role of virulence factors in these three closely related species. In the following paragraphs, a description of functional studies to interrogate gene function will be provided. Figure 1 and Table 1 provides an overview of the genetic manipulation techniques used for the *C. parapsilosis* species complex and the targeted virulence and drug resistance genes.

### 3.1. Lipases

Fungal lipases are secreted hydrolytic enzymes able to catalyze the hydrolysis or synthesis of triacylglycerol [76]. In addition to their important role in nutrient acquisition, lipases are believed to participate to a wide range of additional crucial activities involved in *Candida* infection, such as adhesion to host cells, damage of host tissues, interaction with immune cells and lysis of competing microflora [77,78,79]. Two lipase genes, named *CpLIP1* and *CpLIP2,* and containing a GXSXG motif, usually present in the active sites of most lipolytic enzymes, were identified in *C. parapsilosis* by Neugnot and colleagues in 2002 [40]. Amino acid sequence alignment revealed a high degree of homology with the *C. albicans* lipase gene family, which, in contrast, encompasses 10 members (*LIP1*-*10*). Different gene manipulation approaches were employed over the years with the aim of characterizing lipase function (summarized in Figure 1a). First attempts of gene function studies of *C. parapsilosis* lipases involved the *CpLIP1*-*2* heterologous expression in *S. cerevisiae* [40]. Each *CpLIP* gene was cloned inside an expressing vector and placed under the control of the constitutive alcohol dehydrogenase promoter of *S. cerevisiae.* Transformation and phenotypic characterization of the resulting overexpression *S. cerevisiae* strains indicated lipolytic activity only for *CpLIP2* but not for *CpLIP1*. *CpLIP2* catalytic behavior was additionally observed two years later with its overexpression in the yeast *P. pastoris* [39]. As already mentioned in the previous section, studies performed in *S. cerevisiae* or in any phylogenetically distant yeast have also to take into account the serine/leucine translation change occurring between these organisms and *Candida* species belonging to the CUG clade. In this respect, differently from *CpLIP2*, sequence analysis of *CpLIP1* revealed two CUG codons whose mistranslation may perhaps account for the missing lipolytic activity. In 2007, Gacser and colleagues presented one of the first evidence of *C. parapsilosis* lipases as fungal virulence factors. In this work, the authors demonstrated that ebelactone B, a specific inhibitor of fungal lipases, significantly protected both the human reconstituted oral and epidermal tissues from *C. parapsilosis* infection [80]. In a follow-up study, *C. parapsilosis* lipase function was investigated in the GA1 clinical isolate via targeted gene deletion using the SAT1-flipper cassette [62]. By exploiting the consecutive localization of *CpLIP1* and *CpLIP2* on chromosome 7, the authors targeted the entire lipase locus resulting in the generation of a double mutant strain. A reconstituted strain, obtained by reintroducing a copy of *CpLIP2* in its original genomic location, was generated and included in the analysis. A comprehensive phenotypical characterization of the mutant collection brought to light the key role of lipases in *C. parapsilosis* virulence and pathogenesis. First, deletion of *CpLIP* genes resulted in abolished lipolytic activity, as demonstrated by the absence of a red-stained region around colonies grown on plates containing rhodamine B and the lack of catalytic activity retrieved in supernatant from homozygous lipase-negative cells grown in lipid-containing media. Compared to the wild type, heterozygous and reconstituted strains, the null mutant strain showed reduced growth in lipid-rich media, impaired biofilm formation, and decreased cell injury when tested in a human endothelial cell line. Attenuation of cell damage and reduced levels of released lactate dehydrogenase (LDH) were observed following infection of the human reconstituted epithelium with the lipase-negative mutant cells. To conclude, deletion of *C. parapsilosis* lipase genes resulted in a more efficient killing by murine macrophages-like cells and reduced pathogenic potential when tested in a murine intraperitoneal infection model. As revealed by two follow-up studies published by the Gacser group, the lipase negative strain was more efficiently phagocytized by both human monocyte derived dendritic cells and primary human macrophages and induced higher gene expression of inflammatory mediators than wild type yeasts [23,63]. Following whole-genome sequencing, two additional *CpLIP*-like genes, whose roles to date remain uninvestigated, were suggested to be present in *C. parapsilosis*, [79,81]. Altogether, evidence gathered so far indicates that the role of *C. parapsilosis* lipases is pleiotropic, ranging from enzymes involved in nutrient acquisition, to effector molecules required for biofilm formation, damage of host tissues, and modulation of the immune response.

In contrast, very little is known about lipase production in *C. orthopsilosis* and *C. metapsilosis* and no gene manipulation studies dissecting their role have been published yet. Notably, in 2013 a study aimed at comparing the virulence properties of 93 clinical isolates belonging to the *C. parapsilosis* species complex, most of *C. orthopsilosis* and all *C. metapsilosis* strains failed in producing lipolytic activity, which may partially explain the reduced virulence of the latter member of the complex compared to *C. parapsilosis* [82]. However, sequencing of the *C. orthopsilosis* strain MCO456 indicated a duplication of the lipases locus, resulting in four genes rather than two, as observed in the reference strain 90–125 [83]. Similarly, sequencing of a panel of eleven *C*. *metapsilosis* clinical isolates from different geographical locations revealed the presence of similar number (5) of putative lipase encoding genes [60]. Despite previous studies reporting poor or absent lipolytic activity in *C. orthopsilosis* and *C. metapsilosis* [82], the presence of additional lipase genes retrieved from both genomes suggests that a tuned regulation, possibly depending on the stage of the infection, or the extracellular environment, may affect the expression of lipase genes in these two species, as already observed for *C. albicans* lipases [78]. As a result, gene manipulation studies coupled with specific experimental conditions that can activate the lipase gene expression are required to shed light on the role of *C. metapsilosis* secreted hydrolytic enzymes.

### 3.2. Secreted Aspartyl Proteinases

Secreted aspartyl proteinases (Sap) play a critical role in the pathogenicity of *Candida*. The number of Sap encoding genes varies among *Candida* species, with *C. albicans* possessing 10 genes (SAP1-10), and C. parapsilosis encompassing only three genes, named *SAPP1-3* [84,85], where *SAPP1* consists of an internal duplication, referred to as *SAPP1a* and *SAPP1b* [65]. Only the role of the latter genes has been investigated, despite genome mining analysis performed by Parra-Ortega in 2009 revealing the presence of an additional 11 putative *SAPP* genes of *C. parapsilosis*, for a total of 14 members [86]. First synthetized as pre-pro-enzymes, C. parapsilosis aspartyl proteinases undergo auto-activation or proteolytic processing operated by Kex2-like proteins before being released as mature protein [87]. Among *Candida* species, mature aspartyl proteinases share a common basic structure encompassing an amino terminal signal peptide, four cysteine residues involved in the maintenance of the tridimensional structure and two aspartyl proteases active sites responsible for the catalytic activity [86]. In addition to the simple role of providing nutrients, Sap proteins are considered key enzymes that contribute to the fungal infection by mediating the interaction with the host during the infection and promoting the evasion from the host immune response. C. albicans Sap proteins are extremely versatile: among them we find enzymes that cleave complement proteins like C3b, C4b, and C5 [88], degrade host cell surface structures, enhance fungal attachment to mucosal surfaces [89], inhibit antimicrobial peptides such as histatin-5 [88], or hydrolyze host defense molecules [90,91,92,93]. Similarly, substantial efforts have been made to characterize *C. parapsilosis SAPP* genes. The importance of Sapp proteins in the pathogenicity of *C. parapsilosis* was already evident when the use of the proteinase inhibitor, pepstatin A, dramatically reduced the ability of *C. parapsilosis* strains to cause damage in reconstituted human tissues models [80]. The proteolytic activity of SAPP2 was initially investigated via heterologous expression in *Escherichia coli*, as demonstrated by hemoglobin cleavage test [64]. Figure 1b summarizes all the manipulation techniques employed to dissect the role of Sapp. The first gene manipulation study aimed at dissecting the role of *SAPP* genes was published by Horvath and colleagues with the generation of SAPP1a and SAPP1b single and double mutant strains [65]. Two different SAT1-flipper disruption cassettes, targeting *SAPP1a* and *SAPP1b*, respectively, were generated and transformed in *C. parapsilosis* GA1 clinical isolate. Despite previous reports indicating higher production of *SAPP1* compared to *SAPP2* under inducing conditions [64,94], increased transcripts and protein levels of *SAPP2* were detected when the double knockout strain (ΔΔsapp1a-ΔΔsapp1b) was tested under the same experimental conditions. These data suggested a fine regulation of the *SAPP* gene family and a compensation attempt operated by the double mutant strain. Characterization of the mutant collection revealed a significant reduction of the ΔΔsapp1a-ΔΔsapp1b mutant growth in intact human serum, suggesting a potential role for *SAPP1* in inactivating the host defense mechanisms during *C. parapsilosis* infection, similar to that observed with C. albicans Saps [88]. This observation was further confirmed by the increased killing efficiency of peripheral blood mononuclear cells (PBMCs) and PBMC-derived macrophages (PBMC-DM), and in the increased incidence phagolysosomal fusion PBMC-DMs when challenged with the *SAPP1* defective strain compared to the wild type strain [65].

A comprehensive study aimed at functionally characterizing the role of *SAPP* gene family was published in 2019 [22]. Using the SAT1-flipper cassette, a triple *SAPP* mutant strain was generated in the background of the ΔΔsapp1a-ΔΔsapp1b mutant strain by sequentially deleting *SAPP2* and *SAPP3* genes, respectively. The contribution of individual *SAPP* genes was evaluated through the generation of single reconstituted strains, obtained by reintegrating in the *CpNEUT5L* locus the respective *SAPP* open reading frame under the control of a constitutive promoter (*CaTDH3*). The generation of all reintegrated mutant strains was established in the background of the triple mutant strain in order to avoid any possible cross-interference caused by the remaining members of the *SAPP* family. Characterization of the mutant strains indicated that all *SAPP* genes are involved in the adhesion of *C. parapsilosis* to polystyrene surfaces, while only *SAPP1-2* are required for the adhesion to human oral epithelial cell line, TR146. Similarly, *SAPP1* and *SAPP2*, but not *SAPP3*, were found to contribute to the host cell damage, cytokines production and phagosome-lysosome maturation when their respective reconstituted strains were co-incubated with PBMC-DMs. Finally, purified Sapp1p and Sapp2p proteins were also shown to efficiently cleave human complement proteins, C3b, C4b, complement regulator factor H (FH) and FHR5. Although reintegration in the *CpNEUT5L* locus has been demonstrated to be compatible with knock-in experiments [44], we can’t exclude that the ectopic expression of *SAPP* genes under the control of a constitutive promoter may have an effect on the phenotype of the resulting reconstituted strain, which does not reflect the normal cellular environment. Circumvention of this problem can be achieved through the integration of the desired open reading frame in the original locus, which in turn, however, is more laborious and time-consuming.

When *C. orthopsilosis* and *C. metapsilosis* Sap production was analyzed, a variable proportion of isolates exhibited proteinase activity, which ranged from high protease activity in all *C. metapsilosis* and most of the *C. orthopsilosis* strains, as observed by Németh and colleagues [82], to low, as reported by Sabino [95] and Trevino-Range [96]. In a study performed on 22 strains of *C. metapsilosis* (20 clinical isolates and 2 ATCC reference strains), only five clinical isolates showed secreted proteolytic activity [97]. Interestingly, as reported by the authors, a significant correlation was found between the protease producer strains and their geographic origin [97]. Sequencing of *C. orthopsilosis* and *C. metapsilosis* genomes revealed the presence of 11 and 14 secreted aspartyl proteinases encoding genes [60], respectively, which is more than other *Candida* spp. species, *C. albicans* included. Despite that, the role of *SAPP* genes and their contribution to the virulence of these two species remains to be elucidated.

### 3.3. Phospholipases

Phospholipases are a class of enzymes capable of hydrolyzing one or more ester linkage in glycerophospholipids [98]. These secreted enzymes play an active role during fungal infection, helping the disruption of cell membranes and facilitating host invasion. Although the contribution of *C. albicans* phospholipases were already suggested through the generation of null mutant strains and their subsequent test in various murine models of infections [98,99], the role of this important class of secreted enzymes in the pathogenicity of the *C. parapsilosis* species complex remains to be elucidated. Several studies published throughout the years have reported prolific phospholipase production in *C. parapsilosis* and *C. metapsilosis* from various clinical sources, e.g., blood clinical isolates, and oral and cutaneous samples [100,101,102]. In contrast, no *C. orthopsilosis* strains were included in the analysis and therefore could not be tested [103]. In a work focused on the evaluation of the enzymatic activity of the *C. parapsilosis* species complex, a higher phospholipase activity was displayed by *C. orthopsilosis* strains compared to the other two members of the complex, which in addition statistically correlated with the hematogenous origin of the isolates [96]. Syntenic sequences of the *C. albicans* phospholipase genes, such as *PLB2*, *PLC1*, and *PLD1*, were found in all members of the *C. parapsilosis* species complex, while *PLB1* syntenic gene was retrieved in *C. parapsilosis* only. No genetic manipulation studies have ever been performed even though they will be necessary to clarify the relationship between phospholipases and virulence of the *C. parapsilosis* species complex. 

### 3.4. Adhesion and Biofilm

Adhesion of *Candida* spp. to host surfaces, such as endothelial and epithelial cells, represents a prerequisite for the invasion and the establishment of fungal infection. As well as adhering on biotic surfaces, *Candida* spp. can bind inert materials, such as catheters and prosthetic devices, or interact with the human microflora or other *Candida* species, leading to the formation of surface-associated microbial communities, known as biofilms, which are characterized by a reduced susceptibility to antifungal drugs [104]. Because of the above-mentioned reasons, adhesion has been recognized as a major virulence factor. Compared to *C. parapsilosis* and *C. orthopsilosis*, virulence studies available so far have identified *C. metapsilosis* as the least virulent member, either in terms of colonization/invasion of the reconstituted epithelium [80] or with regard to the susceptibility to phagocytosis and anticandidal activity in an in vitro infection model of microglial cells [105]. Despite *C. parapsilosis* strains produced quantitatively less biofilm with reduced structural complexity compared to *C. albicans* [81,106], a positive correlation between biofilm-forming isolates and increased mortality was observed, as shown in a retrospective 5-year period cohort study performed in an Italian hospital [107]. When biofilm formation is analyzed, *C. parapsilosis* clinical isolates tend to be more prolific biofilm producers than *C. orthopsilosis* and *C. metapsilosis* [3,97,108,109], a characteristic that seems to be reflected on their adhesive ability. In fact, assessment of the adhesive properties of reference and clinical isolates belonging to the three species, indicated similar adhesion abilities for *C. parapsilosis* and *C. orthopsilosis*, while a significantly reduced adhesion to human buccal epithelial cells (HBECs) was displayed by *C. metapsilosis* [10]. A large repertoire of adhesins, localized at the cell wall level mostly via a GPI anchor, have been identified in all three species of the *C. parapsilosis* species complex, potential contributing to fungal adhesion and biofilm formation. These adhesins are mainly organized in three gene families: (i) the agglutinin-like sequence (*ALS*) gene family; (ii) the IPF family F/hyphal-upregulated protein (*IFF/HYR*); (iii) the hyphal wall protein (*HWP*) gene family [21,59,104,110]. A recent comprehensive study on the cell wall proteome of *C. parapsilosis* identified members of all three gene families in the cell wall extracts of clinical isolates [109].

The *ALS* gene family, originally discovered in *C. albicans* more than two decades ago [108,111], and later identified in all of the three members of the *C. parapsilosis* species complex [20,21,59], encodes for cell wall glycoproteins involved in the adhesion to both biotic and abiotic surfaces. Cross hybridization studies between *C. albicans ALS* genes and the genome of the *C. parapsilosis* CDC317 sequenced strain revealed the presence of 5 *ALS* genes (*CpALS4770*, *CpALS4780*, *CpALS4790, CpALS4800,* and *CpALS0660*) [21,59]. By contrast, 3 *ALS* genes (*CoALS4210*, *CoALS4220*, *CoALS800*) were identified in the sequenced strain of *C. orthopsilosis* 90–125 [61], whose sequences, however, were initially incomplete due to the presence of extensive tracts of repeated sequences which are known to be difficult to assemble from short-read sequence data. A new assembly obtained with the joint use of Illumina (short-read) and Oxford Nanopore (long-read) methods recently allowed the sequencing of misassembled repetitive regions and confirmed the number of *ALS* genes [20]. The use of the same sequencing approach revealed 4 *ALS* genes (*CmALS4210*, *CmALS4220*, *CmALS800,* and *CmALS2265*) in *C. metapsilosis* [21]. Structure analysis of the Als proteins indicated that just a small portion of the *C. parapsilosis* species complex adhesins fits in the “NT/T/TR/CT” consensus definition proposed for *C. albicans* adhesins [108]. This model envisioned Als proteins as organized in an amino terminal (NT) domain, mainly involved in the adhesion process, followed by a threonine rich (T) region, a tandem repeat and a Ser/Thr rich C-terminal (CT) domain. In contrast, the majority of the Als proteins of the *C. parapsilosis* species complex lack a TR region, which is instead replaced with imperfect repeats (SSSEPP motif and/or a GSGN+ motif) [21].

Figure 1c recapitulates the gene disruption and gene editing techniques used to dissect the role of adhesins and transcription factors in adhesion and biofilm formation in the *C. parapsilosis* species complex. The first evidence of a direct role of *ALS* genes in the ability of *C. parapsilosis* to adhere to host surfaces was performed by Bertini and colleagues in 2016, with the generation of a *CpALS4800* mutant collection encompassing heterozygous, null mutant and complemented strains using the SAT1-flipper cassette [66]. Characterization of the *CpALS4800* null mutant strain indicated a marked reduction in the adhesion ability to HBECs and in the pathogenic potential if tested in a murine model of urinary infection. The introduction of a wild type allele in the native genomic locus restored the wild type phenotype, confirming the direct role of *CpALS4800* in the adhesion process of *C. parapsilosis*. Both the wild type and null mutant strains obtained in the previous study were tested by Neale and coworkers for their ability to bind immobilized host extracellular matrix proteins under physiological fluid shear forces, which are typically encountered when *Candida* reaches the bloodstream. Results obtained further supported the previously reported role of *CpALS4800* in *C. parapsilosis* virulence [67]. Moreover, heterologous expression studies performed by the same authors indicated increased adhesion of *S. cerevisiae* under the same shear forces [67]. Similar findings were reported with the SAT1-flipper mediated disruption of *CpALS4790* and *CpALS0660*, showing the contribution of only *CpALS4790* in the adhesion to HBECs. The adhesion assay on either HBECs or human umbilical vein endothelial cells (HUVECs) (data not shown), did not show any reduced adhesion of the *CpALS0660* mutant strain, suggesting that further investigation aimed at understanding the adhesion role of this gene is required. Interestingly, both *CpALS4790* and *CpALS0660* contributed to *C. parapsilosis* pathogenesis when tested in a murine model of vaginal infection [68]. Difficulties in the deletion of the remaining wild type allele of *CpALS0660* were overcome with the generation of an alternative disruption cassette targeting a more internal upstream homology region. Similar difficulties in generating homozygous mutants using the conventional gene disruption approaches were already described by Zhao and colleagues when *ALS2* of *C. albicans* was selected as target for mutagenesis [112]. The high efficiency of the CRISPR/Cas9 technology in generating homozygous mutations with unprecedented ease and accuracy, was harnessed by our group to investigate the role of the remaining two uncharacterized *C. parapsilosis ALS* genes, *CpALS4770* and *CpALS4780* [69]. Single and double mutant strains lacking functional Als proteins were readily generated using the episomal CRISPR/Cas9 technology, described by Lombardi and colleagues [55], and a repair template carrying two stop codons and a restriction site. The mutant collection was generated in the background of CP50, a clinical isolate of *C. parapsilosis* previously characterized as highly adherent to HBECs [10]. Phenotypical characterization of the mutant strains unveiled the key role of *CpALS4770* in biofilm formation on polystyrene surfaces and adhesion to HBECs. Notably, the double mutant strain showed an increased ability to adhere to HBECs, a peculiar characteristic that was also observed when biofilm formation was assessed. The exposure of different adhesive moieties may account for the hyper-adhesive phenotype of the double mutant strain, as already hypothesized for other *C. albicans ALS* mutant strains [113]. Conversely, both single and contextual deletion of *CpALS4770* and *CpALS4780* genes negatively affected the ability of *C. parapsilosis* to colonize and persist in the host environment, as highlighted from the murine vaginal infection experiment [69].

While to date no genetic manipulation studies have been performed to decipher the role of *ALS* genes in *C. metapsilosis*, substantial efforts have been made in the closely related species, *C. orthopsilosis*. A gene disruption approach based on the SAT1-flipper cassette was used by our group to dissect the role played by the *C. orthopsilosis CoALS4210* gene [47]. Heterozygous and null mutant strains were obtained in the background of the sequenced strain, 90–125. In the same study, two independent CRISPR/Cas9 edited strains, lacking a functional CoAls4210 adhesin, were generated to validate the phenotype observed in the homozygous gene disrupted strain, demonstrating at same time the adaptability in *C. orthopsilosis* of the episomal CRISPR/Cas9 technology described by the Butler group [47]. Both the knockout and gene edited *CoALS4210* strains showed a marked reduction in the adhesion to HBECs, attesting for the first time the involvement of an *ALS* gene in the adhesion process of *C. orthopsilosis*. As previously mentioned, one of the most attractive features of the CRISPR/Cas9 technology is the possibility of targeting, with unprecedented ease, an entire gene family, or at least several members of the same gene family, in a single experiment, thus speeding up enormously the creation of multiple mutant strains. By taking advantage of the high degree of homology shared among the N-terminal domains of *ALS* genes, we designed a unique gRNA able to target each member of the *ALS* gene family of *C. orthopsilosis* [71]. A common repair template was co-transformed and integrated at the Cas9 DSB cutting site, allowing the simultaneous interruption of the open reading frame of *CoALS4210*, *CoALS4220*, *CoALS800* genes with the insertion of premature stop codons. Triple edited strains were obtained in the background of both the sequenced strain, 90–125, and in the highly adhesive clinical isolate, CP124. Both mutant strains showed a dramatic impairment in the adhesion to HBECs, which was even more pronounced for the clinical isolate. Compared to traditional gene disruption techniques, which would have hampered the study of the *ALS* gene family by requiring laborious cloning steps and several transformation and recycling experiments, the CRISPR/Cas9 system allowed us to generate multi-gene edited strains of *C. orthopsilosis* cost-efficiently and in an extremely short amount of time. Moreover, a fungal model in which to investigate the role of single *ALS* genes of *C. orthopsilosis* was generated, also thus allowing the elimination of the cross-interference that other members of the same family may cause.

A plethora of different proteins localized at the cell-wall level is involved in the adhesion of *Candida* spp. to biotic or abiotic surfaces. Another gene family that has been discovered to be enriched in the genome of pathogenic *Candida* species is the Iff/Hyr gene family, which in *C. parapsilosis* encodes for 17 putative genes [21,59]. Studies in *C. albicans* have revealed a role for the Iff/Hyr proteins that goes beyond fungal adhesion to host surfaces [114] and that also involves their participation in the cell wall structural support, as proved by the significant structural alteration displayed by the *IFF11* mutant strain [115]. Interestingly, the *C. parapsilosis* genome shows duplications of members of this gene family on three chromosomes, suggesting a potential role for the Iff/Hyr proteins in the pathogenicity of this species [59]. This is especially true if considered that the closely related species *C. orthopsilosis*, which has been associated with decreased pathogenic potential, does not show any duplication and encompasses only three members [61]. In contrast, 13 *IFF*/*HYR* genes have been identified in *C. metapsilosis* [21,60] and evidences of recombination events between this gene family and the *ALS* genes are supported by the structural analysis of the *CmALS2265* gene, which shows repeated sequences and a C-terminal region resembling the *C. albicans IFF*/*HYR* genes [21]. Unfortunately, no genetic manipulation studies have been performed to date to dissect the role of this gene family in any of the members of the *C. parapsilosis* species complex. This would be of extreme interest in order to explain the apparent incongruence between the reduced pathogenicity of *C. metapsilosis* and its expansion of the *IFF/HYR* gene family compared to *C. orthopsilosis*. Interestingly, *C. metapsilosis* is the only member of the *C. parapsilosis* species complex unable to form pseudohyphae. This defect in morphogenesis appears to correlate with a lower number of *ALS* gene members but not with the *IFF/HYR* gene family [21]. Nevertheless, we believe that the availability of efficient gene editing tools, such as the CRISPR/Cas9 system, will stimulate the *Candida* scientific community to shed light on the role played by *IFF*/*HYR* gene family.

Despite evidence reporting a role for *HWP* genes in the *C. albicans* adhesion to host cell surfaces and biofilm formation, genetic manipulation studies dissecting the role of this gene family in the *C. parapsilosis* species complex is still lacking. Putative homologs of *C. albicans HWP1* and *RBT1*, both belonging to the hypha specific *HWP* adhesin family [116], have been identified in *C. parapsilosis* and *C. orthopsilosis* [104,110]. A syntenic sequence of both *HWP1* and *RBT1* can be retrieved in *C. metapsilosis* in the Candida Gene Order Browser (http://cgob.ucd.ie/ (accessed on 20 April 2021) [117,118]), unlike *HWP2*, which is apparently missing in all three members of the *C. parapsilosis* species complex. The presence of the Rbt1 protein at the cell-wall level has been recently shown by the cell wall proteome studies performed on both *C. parapsilosis* CDC317 strain and clinical isolates [109].

In addition to the role of the adhesion molecules discussed above, other virulence factors, such as transcription factors and other proteins participate to the formation of *C. parapsilosis* biofilms. The *C. albicans BCR1* gene encode for a zing finger protein strictly involved in biofilm formation, as demonstrated by the defective biofilm formed in vitro by *C. albicans* knockout strains [119]. This transcription factor regulates the expression of cell surface proteins, adhesins, such as *ALS3* and *HWP1* genes, as well as the chitinase *CHT2* gene [119,120,121]. The first *C. parapsilosis CpBCR1* deletion collection encompassing heterozygous, knockout and reconstituted strains were generated with the SAT1-flipper cassette using the ATCC 22019 reference strain as parental strain [42]. Unlike the wild type, heterozygous and reconstituted strains, the *CpBCR1* null mutant failed to produce biofilm on silicone surfaces. Despite the fact that deletion of *CpBCR1* did not result in altered *ALS* genes expression profiles, downregulation of *CpRBT1*, a close relative of *C. albicans HWP1*, was shown in the knockout strain [42], which may correlate with the defective biofilm phenotype observed. Interestingly, the *CpBCR1* role as major regulator of *C. parapsilosis* biofilm formation was shown to be strain dependent, as demonstrated by the work of Pannanusorn and colleagues [70]. In this study, *CpBCR1* deletion mutants were obtained in the background of clinical isolates previously characterized as low or high biofilm producers. Surprisingly, the transcription factor CpBcr1 showed to play an essential role in *C. parapsilosis* biofilm formation only for those strains with low capacity for biofilm formation [70]. The phenotypical screen of the collection of *C. parapsilosis* mutant strains created by Holland and colleagues [50] allowed the identification of seven transcription factors (*CpEFG1*, *CpCZF1*, *CpGZF3*, *CpUME6*, *CpCPH2*, *CpBCR1,* and *CpACE2*) and one protein kinase (*CpMKC1*) that are required for in vitro biofilm formation. Interestingly, when biofilm development was tested in a rat central venous catheter model of infection, only some of these genes were found to be required, suggesting a context dependent regulation of *C. parapsilosis* biofilms. As shown by the same authors, the generation of *C. albicans* null mutant strains lacking the orthologous transcription factors and protein kinase found to be major biofilm regulators in *C. parapsilosis*, indicated that only *BCR1*, *EFG1* and *ACE2* show a conserved role in the biofilm regulation between the two species. Thus, *CpCZF1*, *CpGZF3*, *CpUME6*, *CpCPH2,* and *CpMKC1* appear to be unique regulators of *C. parapsilosis* biofilm development pathways.

## 4. Drug Susceptibility in *Candida parapsilosis* Species Complex

The emergence and continuous rise of antifungal resistance and multi-resistance represents an important threat to the patient management and clinical success, as this drastically reduces the treatment options [122]. The incidence of antifungal resistance among *C. parapsilosis* species complex has been kept at low and at non-alarming numbers for many years as shown by surveillance studies [11,123]; however, late reports outlined an increasing isolation rate of isolates resistant to antifungal drugs whose occurrence varies among different geographic regions [18,124,125].

Four classes of antifungal drugs: azoles, echinocandins, polyenes, and nucleoside analogs are available for candidemia treatment [126]. Fluconazole was first introduced in general practice and it is still widely used in prophylaxis [126,127]. Nowadays, depending on the clinical presentation, echinocandins, or amphotericin-B are recommended as first-line treatment for invasive and disseminated candidiasis; however, fluconazole is still suitable for non-critically ill patients [126]. It is believed that this extensive use of fluconazole is associated with increased incidence in azole resistance among non-albicans *Candida* species [128].

Molecular mechanisms of antifungal resistance have been widely characterized for *C. albicans*, but little is known for the members of the *C. parapsilosis* species complex [129,130]. Recognized azole resistance mechanisms in *C. albicans* include (a) overexpression of ATP-binding cassette (*ABC*) superfamily drug transporters *CDR1*, *CDR2* and major facilitator superfamily (MFS) *MDR1* transporter; (b) gain of function mutations in the *TAC1* and *MRR1* gene transcription factors associated to the overexpression of the mentioned efflux pumps of the *ABC* and *MFS* superfamily, respectively; (c) overexpression of *ERG11* associated to the presence of gain of function mutations in the transcription factor Upc2; (d) drug target alteration associated with the presence of amino acid substitutions in Erg11, resulting in a lower drug binding affinity; and (e) inactivation of Erg3 [129,131]. Echinocandin resistance in *C. albicans* is associated with the presence of point mutations in the *FKS1* gene that encodes a subunit of the β-(1,3)-glucan synthase [132].

Studies to elucidate the resistance mechanisms in these species have been mainly assessed using orthologous of *C. albicans* genes known to be associated with antifungal resistance. Strategies such as whole genome sequencing (WGS), sequencing, and expression analysis have been commonly used to characterize the resistant phenotype. However, a complete validation of the ascribed mechanisms in the development of resistance by means of gene manipulation strategies has been afforded by few authors. In the following sections, an overview of the study of antifungal resistance using different gene manipulation strategies among the members of the *C. parapsilosis* species complex will be presented (Figure 1c).

## 5. Genetic Manipulation Approaches to Investigate *C. parapsilosis* Species Complex Drug Resistance

### 5.1. Azole Resistance in Candida parapsilosis Species Complex

Azole drugs, among them fluconazole, voriconazole, posaconazole, itraconazole, and isavuconazole are heterocyclic synthetic compounds that target the cytochrome P450 enzyme lanosterol-14α-demethylase involved in the biosynthesis of ergosterol, a major component of the fungal cell membrane [133]. Lanosterol-14α-demethylase is encoded by the *ERG11* gene in *Candida* species and its inhibition exerts a fungistatic effect as this leads to the disruption of the cell membrane stability and permeability, irregular function of membrane-associated proteins and the accumulation of toxic sterol intermediates like 14-α-methyl-3,6-diol [133,134].

Azole resistance among *C. parapsilosis* species complex has been described worldwide [135]. Assessment on the underlying molecular mechanism associated with antifungal resistance has unveiled the resemblance to some of the already known drug resistance mechanisms in *C. albicans*, as well as the potential role of different mechanisms not previously described in this species. Although the number of works describing the association of these different mechanisms to a resistant phenotype are consistent, the validation and characterization using gene manipulation strategies were less explored.

Association of overexpression of *CpMRR1* to fluconazole resistance in *C. parapsilosis* clinical isolates was first suggested by Grossman and collaborators [14]. Similarly, Silva and colleagues demonstrated that overexpression of *CpMDR1* and *CpMRR1* correlated with point mutations in *CpMRR1* in laboratory-induced, azole-resistant *C. parapsilosis* isolates derived from fluconazole and voriconazole in vitro exposure. In addition, the same authors also showed upregulation of two transcription factors *CpUPC2*, *CpNDT80,* and 13 different *CpERG* genes including *CpERG11* in resistant strains exposed in vitro to posaconazole [136]. Validation of these resistance mechanisms was undertaken by the same research group using the SAT1-flipper cassette (Table 1). Branco and coworkers assessed the contribution of the different *CpMRR1* non-synonymous mutations in the development of resistance using in vitro induced fluconazole and voriconazole *C. parapsilosis* resistant isolates [136]. *CpMRR1* alleles containing G1747A, A2619C and A3191C point mutations, leading to the G583R, K873N, and Q1064P amino acid substitutions, respectively, were integrated into a *C. parapsilosis* mutant strain [50] where both copies of the *CpMRR1* had been deleted. Upon acquisition of the gene harboring the point mutations, it was observed that G583R, K873N, but not Q1064P conferred resistance to both fluconazole and voriconazole. Furthermore, expression levels of *CpMRR1* and *CpMDR1* among the mutant strains were assessed and compared to the wild type ones: data obtained indicated that both mutants had higher levels of expression. In particular, *CpMDR1* transcripts showed a 70-fold higher expression level, which further confirmed the role of G583R, K873N amino acid substitutions in *CpMRR1* and *CpMDR1* overexpression as a mechanism of azole resistance [72].

Furthermore, the contribution of overexpression of the transcription factors *CpUPC2* and *CpNDT80* to azole resistance in *C. parapsilosis* was evaluated. As previously mentioned, in vitro exposure to posaconazole resulted in the development of fluconazole, voriconazole, and posaconazole resistance, and was linked to overexpression of *CpUPC2*, *CpNDT80,* and a number of *ERG* genes [136]. Deletion of *CpUPC2* and *CpNDT80* individually or in combination using the SAT1-flipper cassette strategy showed a restoration of azole susceptibility and a decrease in the expression levels of overexpressed *ERG* genes in the resistant isolate, even though in both cases the deletion of *CpUPC2* had a more significant effect. For instance, fluconazole MIC value (<0.125 µg/mL) in the *CpUPC2* knockout mutant strain was lower than the parental susceptible strain (1 µg/mL) [73].

Besides *MDR1, CDR1* and *ERG11* were also found overexpressed in fluconazole-resistant *C. parapsilosis* clinical isolates [74]. Assessment of the individual role of the two drug transporters was performed using the SAT1-flipper cassette gene disruption strategy. Deletion of both alleles led to a reduction in the MIC value (1-dilution decrease susceptibility), but it did not restore the susceptibility. Interestingly, despite *CpMDR1* deletion, one of the mutant strains did not show any susceptibility modification. Although the role of different amino acid substitutions (G650E, L978W) in *CpTAC1* and (A854V, R479K, I283R) in *CpMRR1* was not evaluated in this study, their association to the overexpression of *CpCDR1* and *CpMDR1* respectively, and the regulation of different proteins contributing to azole resistance was suggested. These results indicated that *CpCDR1* and *CpMDR1* overexpression partially contributes to the azole resistance in *C. parapsilosis*. However, other mechanisms different from the ones commonly found in *C. albicans* are likely to be associated to the development of a drug resistant phenotype in *C. parapsilosis*. It was also determined that activating mutations in the *CpUPC2* transcription factor may not account for the level of overexpression of *CpERG11* [74].

Alteration in the gene target *CpERG11*, such as the A395T missense mutation leading to the amino acid substitution Y132F already described in *C. albicans* was also reported in *C. parapsilosis* fluconazole-resistant isolates [14,74]. Indeed, it is the one most commonly associated with the development of resistance in *C. parapsilosis* [14]. In a recent study, Singh and collaborators described the Y132F amino acid substitution and novel A428G non-synonymous mutation leading to the K143R amino acid substitution, widely distributed among resistant and susceptible-dose dependent (SDD) isolates with a frequency of about 92%. The role of both mutations in the development of resistance was assessed by gap-repair cloning introduced into *S. cerevisiae* (Table 1). It was observed that the presence of the vector containing the mutation led to an increase of resistance compared to the WT *CpERG11* or the empty vector (4- to 16-fold greater MIC values) [16].

Azole resistance in *C. albicans* was also suggested to be associated with the inactivation *ERG3* [131]. In *C. parapsilosis,* two amino acid substitutions R135I [73] and G111R [75] are thought to have a potential role in azole resistance associated with impaired activity of sterol C-5 desaturase encoded by *CpERG3*. Rybak and collaborators assessed the role of Erg3 and an amino acid substitution in the development of antifungal drug resistance in two genetically related *C. parapsilosis* clinical isolates. A strain resistant to azole drugs presenting echinocandin intermediate resistance, and a susceptible strain were investigated. The SAT1-flipper cassette strategy was used first to delete the *CpERG3* in both the azole-resistant and susceptible isolates; interestingly, *ERG3* deletion led to a resistant phenotype to fluconazole and to an intermediate/resistant phenotype to echinocandin drugs. In addition, evaluation of the role of CpErg3 G111R amino acid substitution by the same cassette strategy showed that replacement of both alleles containing the point mutation in the resistant isolate with the WT gene from the susceptible isolate restored the susceptible phenotype. Notably, echinocandin susceptibility was also restored. Moreover, gas chromatography (GC)-mass spectrometry (MS) complementary analysis showed that the presence of the amino acid substitution was associated with a reduced activity of the protein a reduction of the sterol desaturase activity was observed as the accumulation of ergosta-7,22-dienol, and ergosta-7-enol [75].

Regarding *C. orthopsilosis*, few data are currently available to address this point. We characterized a panel of *C. orthopsilosis* clinical isolates from two different geographical regions to assess the frequency and molecular mechanism(s) underlying azole-resistant phenotypes: 16 of 40 isolates were found to be resistant to fluconazole and at least one other azole. Sequencing of *CoERG11* demonstrated the presence of six non-synonymous mutations, five of which were present in both resistant and susceptible isolates, while only the A395T mutation Y132F amino acid substitution occurred among resistant strains (10 out of 16 resistant isolates). The effect of the A395T mutation on *C. orthopsilosis* susceptibility to azoles was evaluated using the SAT-1 flipper cassette strategy. A WT copy of *CoERG11* was replaced by a gene copy containing the A395T mutation, and both homozygous and heterozygous mutants were successfully obtained. The presence of the heterozygosis mutation was sufficient alone to induce azole multi-resistance, with the homozygous mutant showing the highest MIC values [19].

In a recent study, CRISPR-Cas9 technology was applied to evaluate the role of two amino acid substitutions in *CoERG11* gene as a mechanism of azole resistance in *C. orthopsilosis*. From a fluconazole-resistant clinical strain in which six different heterozygotic polymorphisms were found, two mutations (T1126A and G1372A, leading to the L376I and G458S amino acid substitutions, respectively) were further investigated as likely to explain the resistant phenotype. Single base editing was performed to introduce the desired mutation in a fluconazole susceptible *C. orthopsilosis* isolate. It was demonstrated that G458S, but not L376I, had a role in the development of fluconazole and voriconazole resistance, while no effect in posaconazole susceptibility was observed. Heterozygous and homozygous mutants were obtained for G458S. The fluconazole MIC in heterozygous mutants showed a four-fold increase from 0.5 to 2 µg/mL, while in homozygous mutants an eight-fold increase (4 µg/mL) was observed [28]. No studies addressing the molecular basis of isavuconazole resistance have been performed, thus preventing a full comprehension on azole resistance in these species.

While azole resistance is generally uncommon in *C. metapsilosis*, some studies have reported the isolation of resistant or dose dependent susceptible isolates [137,138]. In this species, no gene manipulation studies to unveil the antifungal resistance mechanisms are currently available.

### 5.2. Echinocandins Resistance in Candida parapsilosis Species Complex

Echinocandins drugs are natural product derivatives composed of a cyclic hexapeptide core with an N-linked fatty-acyl side chain: caspofungin, anidulafungin, and micafungin are fungicidal drugs that bind Fks1, one of the catalytic subunits of (1,3)-β-D-glucan synthase involved in cell wall biosynthesis [139]. The inhibition of (1,3)-β-D-glucan synthesis leads to a disruption of the cell wall integrity and the imbalance in osmotic pressure [129]. A unique feature among *C. parapsilosis* species complex is the intrinsic reduced in vitro susceptibility to echinocandins as a result of a naturally occurring polymorphism non-synonymous mutation P660A in *FKS1* [140]. Echinocandin resistance in *C. parapsilosis* is not common, indeed a 0.1% frequency has been reported in a 20-year surveillance study including 135 medical centers in different countries of North America, South America, North America, Latin America, Europe, and the Asia-Pacific region [125]. Only one gene manipulation study has assessed echinocandin resistance in *C. parapsilosis*, with the Erg3 G111R amino acid substitution observed [75] as described above. To the best of our knowledge, no studies regarding *C. orthopsilosis* or C. *metapsilosis* have been performed yet.

## 6. Conclusions

Current knowledge on the complex interplay between the fungal species *C. parapsilosis*, *C. orthopsilosis*, *C. metapsilosis* and the host is still incomplete. As emerged from this review, most evidence was collected on *C. parapsilosis*, while *C. metapsilosis*, the least virulent member of the complex, remains a poorly characterized yeast, despite being capable of causing serious infections in the clinical setting. Identification of virulence factors and molecular mechanisms associated with antifungal resistance have been afforded, mainly thanks to functional studies, where gene disruption and more recently gene editing strategies have allowed characterization of new genes and their association with virulence or antifungal resistance. Moreover, in light of the emergence of multidrug resistance strains, the selective targeting of virulence mechanisms represents a valid alternative to the overuse of conventional antifungal drugs. Anti-virulence drugs were not only shown to be less toxic, but also less prone to induce resistance, compared to conventional antifungal therapies [141]. Among the virulence factors, adhesion of *Candida* spp. to biotic surfaces is a necessary prerequisite for host colonization and infection. As a result, the development of new molecules inhibiting or preventing the adhesion process holds great promise as a novel therapeutic strategy, with some compounds targeting *C. albicans* Als currently undergoing preclinical studies and clinical trials [141,142,143,144].

We need to expand upon these novel therapeutic strategies to other fungal pathogens, but to do so, much groundwork still needs to be performed on NAC species. Effectiveness and flexibility of CRISPR/Cas based toolbox has the potential to significantly speed up genetic manipulation of pathogenic fungi. Indeed, similar to the deletion libraries generated in *C. albicans* [145,146], the unprecedented ease and precision of the CRISPR technology in gene targeting has the potential to be further exploited and upgraded for high-throughput functional studies in the *C. parapsilosis* species complex. This will consequently allow the large-scale generation of mutant strains and the broadening of the current knowledge on virulence and drug resistance traits of these species.

## Figures and Tables

**Table 1:** Overview of the genetic manipulation studies dissecting *C. parapsilosis* species complex virulence factor and summarized outcome of the phenotypical characterization.

Virulence Factor	Species	Genes	Genetic Manipulation Technique Used	Phenotypical Characterization
Lipases	*C. parapsilosis*	*CpLIP1-2*	Heterologous expression in *S. cerevisiae*	Lipolytic activity detected only for *CpLIP2* but not for *CpLIP1* [40].
		*CpLIP2*	Heterologous expression in *P. pastoris*	*CpLIP2* lipolytic activity detected [39].
		*CpLIP1-2*	SAT1-flipper cassette	CpLip1/2^−/−^ mutant strain exhibit abolished catalytic activity, reduced growth in lipid-rich media, impaired biofilm formation, more efficient killing by macrophages-like cells and monocyte derived dendritic cells, and reduced pathogenic potential in a murine intraperitoneal infection model [23,62,63].
Secreted aspartyl proteinases	*C. parapsilosis*	*SAPP2*	Heterologous expression in *E. coli*	*SAPP2* proteolytic activity was demonstrated through the hemoglobin cleavage test [64].
		*SAPP1a-b*	SAT-1 flipper cassette	Sapp1a/1b ^−/−^ mutant strains showed Sapp2 overexpression under induced conditions, growth reduction in human serum, increased killing by PBMCs and PBMC-DM and increased phagolysosomal fusion in PBMC-DMs [65].
		*SAPP1a-b*, *SAPP2*, *SAPP3*	SAT-1 flipper cassette	A sapp1/2/3^−^^/−^ defective strain was generated as well as reintegrated mutants. All *SAPP* genes are involved in the adhesion to polystyrene surfaces; *SAPP1* and *SAPP2* are required for the adhesion on TR146 cells and host cell damage, phagocytosis, phagosome-lysosome maturation, killing, and cleavage of human complement proteins [22].
Adhesion and biofilm	*C. parapsilosis*	*CpALS4800*	SAT1-flipper cassette	Marked reduction of the *CpALS4800* null mutant strain in the adhesion to HBECs and in the pathogenic potential if tested in a murine model of urinary infection. Reintegration of *CpALS4800* in the original locus restored the adhesive ability [66].
			Heterologous expression in *S. cerevisiae*	*CpALS4800* expression resulted in the increased adhesion of *S. cerevisiae* [67].
		*CpALS4790*- *CpALS0660*	SAT1-flipper cassette	*CpALS4790* is required for the adhesion to HBECs. Deletion of either *CpALS4790* or *CpALS0660* resulted in a reduced pathogenic potential when tested in a murine model of vaginal candidiasis [68].
		*CpALS4770* *CpALS4780*	CRISPR/Cas9 system	*CpALS4770* edited strain showed impaired ability to form biofilm on polystyrene surfaces and to adhere on HBECs. The contextual deletion of *CpALS4770* and *CpALS4780* resulted in an increased tendency of the double mutant strain to form cellular aggregates, adhere on HBECs, and form biofilm on plastic surfaces. Both single and double mutant strains showed a reduced ability to colonize and persist in the murine vaginal mucosa [69].
		*CpBCR1*	SAT1-flipper	*CpBCR1* is required for *C. parapsilosis* biofilm formation on silicone surfaces and for the expression of the cell wall protein *CpRBT1* [42]. Clinical isolates prolific in biofilm production are not dependent on CpBcr1 transcription factor [70].
		Transcription factors: *CpEFG1, CpCZF1, CpGZF3, CpUME6, CpCPH2, CpBCR1, CpACE2*, Protein kinase: *CpMKC1*	Gene disruption cassette generated by fusion PCR	Gene disruption resulted in impaired biofilm formation in vitro and in vivo [50].
	*C. orthopsilosis*	*CoALS4210*	SAT1-flipper cassette and CRISPR/Cas9 system	*CoALS4210* knockout and CRISPR edited strains showed reduced adhesion to HBECs [47].
		*CoALS410* *CoALS4120* *CoALS800*	CRISPR/Cas9 system	Triple edited strains lacking the entire *ALS* gene family showed dramatic reduction in the adhesion to HBECs [71].
Drug susceptibility	*C. parapsilosis*	*CpMRR1*	SAT1-flipper cassette	Acquisition of point mutations G1747A, A2619C leading to G583R, K873N amino acid substitutions, respectively were involved in the development of fluconazole and voriconazole resistance, in addition to *CpMDR1* and *CpMRR1* overexpression [72].
		*CpUPC2* *CpNDT80*	SAT1-flipper cassette	Deletion of overexpressed *CpUPC2* and *CpNDT80* alone or in combination in fluconazole, voriconazole, and posaconazole-resistant isolates led to the restoration of in vitro susceptibility. *CpUPC2* deletion had a more significant effect [73].
		*CpMDR1* *CpCDR1*	SAT1-flipper cassette	Deletion of overexpressed *CpMDR1* and *CpCDR1* in azole-resistant isolates led to the partial restoration of in vitro susceptibility [74].
		*CpERG11*	Heterologous expression in *S. cerevisiae*	Acquisition of A395T and A428G point mutations leading to Y132F and K143F amino acid substitutions, respectively, were involved in the development of in vitro azole resistance in *S. cerevisiae* [16].
		*CpERG3*	SAT1-flipper cassette	*CpERG3* knockout led to in vitro azole resistance and intermediate resistance to echinocandins. CpErg3 G111R amino acid substitution was involved in in vitro azole and echinocandin resistance [75].
	*C. orthopsilosis*	*CoERG11*	SAT1-flipper cassette	Acquisition of A395T leading to Y132F amino acid substitution, was involved in the development of in vitro azole resistance. Highest MIC values in homozygous mutant [19].
			CRISPR/Cas9 system	G1372A point mutations leading G458S amino acid substitution was involved in in vitro fluconazole and voriconazole resistance. Highest MIC values in homozygous mutants. No effect in posaconazole resistance was observed [28]

## Data Availability

Not applicable.
